# Host Directed Therapies for Tuberculous Meningitis

**DOI:** 10.12688/wellcomeopenres.16474.1

**Published:** 2020-12-23

**Authors:** Angharad G. Davis, Joseph Donovan, Marise Bremer, Ronald Van Toorn, Johan Schoeman, Ariba Dadabhoy, Rachel P.J. Lai, Fiona V Cresswell, David R Boulware, Robert J Wilkinson, Nguyen Thuy Thuong Thuong, Guy E Thwaites, Nathan C Bahr, Tuberculous Meningitis International Research Consortium

**Affiliations:** 1 University College London, Gower Street, London, WC1E 6BT, UK; 2 The Francis Crick Institute, Midland Road, London, NW1 1AT, UK; 3 Wellcome Centre for Infectious Diseases Research in Africa, Institute of Infectious Disease and Molecular Medicine, University of Cape Town, Observatory, 7925, South Africa; 4 Oxford University Clinical Research Unit, Centre for Tropical Medicine, Ho Chi Minh City, Vietnam; 5 Centre for Tropical Medicine and Global Health, Nuffield Department of Medicine, University of Oxford, Oxford, UK; 6 Department of Pediatrics and Child Health, Stellenbosch University, Cape Town, 7505, South Africa; 7 Division of Infectious Diseases, Department of Medicine, University of Kansas, Kansas City, KS, USA; 8 Department of Infectious Diseases, Imperial College London, London, W12 0NN, UK; 9 Department of Clinical Research, London School of Hygiene and Tropical Medicine, London, WC1E 7HT, UK; 10 Infectious Diseases Institute, Makerere University, Kampala, Uganda; 11 Division of Infectious Diseases and International Medicine, Department of Medicine, University of Minnesota, Minneapolis, MN, USA

**Keywords:** Tuberculous Meningitis, Host Directed Therapies, Dexamethasone

## Abstract

A dysregulated host immune response significantly contributes to morbidity and mortality in tuberculous meningitis (TBM). Effective host directed therapies (HDTs) are critical to improve survival and clinical outcomes. Currently only one HDT, dexamethasone, is proven to improve mortality. However, there is no evidence dexamethasone reduces morbidity, how it reduces mortality is uncertain, and it has no proven benefit in HIV co-infected individuals. Further research on these aspects of its use, as well as alternative HDTs such as aspirin, thalidomide and other immunomodulatory drugs is needed. Based on new knowledge from pathogenesis studies, repurposed therapeutics which act upon small molecule drug targets may also have a role in TBM. Here we review existing literature investigating HDTs in TBM, and propose new rationale for the use of novel and repurposed drugs. We also discuss host variable responses and evidence to support a personalised approach to HDTs in TBM.

## Introduction

Clinical outcomes in tuberculous meningitis (TBM) depend upon both killing *Mycobacterium tuberculosis (M.tb)* and managing host inflammatory response. Antimicrobial drug therapy for TBM has been adapted from that used for pulmonary tuberculosis (TB); four drugs are given initially, with subsequent tapering to two or three drugs (in drug-susceptible TBM, dependent on local guidelines) for continuation of therapy up to one year. Yet the host immune response may be dysregulated, and contributes to the poor outcomes associated with TBM. Host directed therapies (HDTs) seek to control this host response and reduce death and neurological injury.

The discovery and assessment of new therapeutics in TBM has been a neglected area; this includes the development of bespoke antitubercular drug regimens that account for differing ability of drugs to penetrate the central nervous system (CNS), and the design of HDTs that counter dysregulated immune responses to *M.tb* within the CNS. In fact, corticosteroids are the only widely used host directed therapy in TBM with any proven benefit in both adults ^1^ and children ^2^. In adults, in particular, questions around their clinical use remain, including whether they have a role in improving outcomes in HIV-associated TBM and the mechanisms by which they improve survival. Clinical trials to assess the efficacy of other HDTs including aspirin and thalidomide have been conducted; however, there is not yet conclusive evidence to suggest when, with whom and at what dose they may be effective. New knowledge from studies uncovering mechanisms of inflammation and brain injury may also allow for a directed approach to modulating the host response. Similarly, studies aiming to contribute knowledge of factors at play that influence variability in the host may lead us away from a ‘one size fits all’ therapeutic approach.

We review the evidence on currently used HDTs in TBM and suggest potential therapeutics based on pathogenesis studies and drawing from knowledge and experience in other forms of tuberculosis and neuroinflammatory conditions. We will review work that has contributed to our understanding of variation in host response and discuss how this knowledge might be harnessed to design a personalised approach to the use of HDT in TBM.

## Existing host directed therapies for tuberculous meningitis

### Dexamethasone

Adjunctive corticosteroids reduce mortality from TBM, at least in the short term ^1, 3, 4^. The mechanism through which corticosteroids confer clinical benefit is unclear, although reduction in intracerebral inflammation seems most likely. Glucocorticoids bind to and activate the glucocorticoid receptor of macrophages and other cells, interfering with inflammatory mediator transcription and expression ^5^. Additional indirect genomic effects of inhibition of pro-inflammatory transcription factors such as activator protein-1, and non-genomic mechanisms further mediate glucocorticoid anti-inflammatory effects ^6– 9^.

Murine studies suggest *M.tb* induces activation of the microglial NLRP3 inflammasome, a multimolecular immune complex of receptors and sensors that mediates innate immune responses and induces inflammation via pro-inflammatory caspases and cytokines; a process inhibited by dexamethasone ^10, 11^. In TBM, pro-inflammatory cerebrospinal fluid (CSF) cytokine concentrations are acutely elevated, although therapeutically reducing these concentrations may not be clinically beneficial. In a study of 16 individuals with TBM in India, concentrations of tumor necrosis factor (TNF)-α, interleukin (IL)-1β, IL-6, IL-8, IL-10 were elevated in TBM vs. controls, and declined during TB treatment, yet cytokine concentrations were not related to disease severity, brain magnetic resonance imaging (MRI) abnormalities or clinical outcome ^12^. In a paediatric study (n=30), CSF TNF-α, IL-1β, and interferon (IFN)-gamma concentrations were elevated in acute TBM, but again did not correlate with disease severity, nor were they influenced by corticosteroid administration ^13^. However, in a large study of clinical and intracerebral inflammatory phenotype and nine-month survival in adults with TBM from Vietnam, multiple pro-inflammatory and anti-inflammatory CSF cytokines were significantly reduced in HIV uninfected individuals who died vs. in HIV uninfected who survived ^14^. This effect (lower pro-inflammatory cytokines in individuals who died) was not seen in HIV co-infection. 

In 545 Vietnamese individuals >14 years recruited to a randomized placebo-controlled trial of dexamethasone for TBM, dexamethasone was associated with a reduced risk of death (relative risk 0.69, p=0.01) ^1^. In a representative subset of this study, dexamethasone did not significantly alter tested CSF cytokines (TNF-α, IL-1β, IL-6, IL-8, IL-10, IL-12) over time vs. placebo ^15^. CSF concentrations of IL-6, IL-8, and IL-10 fell slowly after commencement of anti-TB chemotherapy, and TNF-α fell rapidly, all irrespective of dexamethasone treatment. In a subgroup of HIV uninfected adults (n=37), dexamethasone significantly reduced CSF matrix metalloproteinase-9 (MMP-9) in follow up samples taken after a median five days of treatment ^16^. Further work is required to determine whether the protective effect of dexamethasone with a measurable reduction in intracerebral inflammation.

International guidelines recommend adjunctive corticosteroids for TBM management ^17^. Corticosteroid use in TBM is commonplace, dexamethasone is commonly used as it is affordable and widely available although the optimal corticosteroid preparation, dose, and route of administration are unknown ^18^. Whether beneficial therapeutic effects extend to HIV co-infected individuals is uncertain. In a HIV-positive subgroup (n=98) from a randomized trial of adjunctive corticosteroids for TBM in Vietnamese adults, dexamethasone was associated with a non-significant trend towards improved survival ^1^. Subsequently, a study of adults with HIV-associated TBM showed global increase in pro-inflammatory cytokine concentrations, running counter to theory that immunosuppressed HIV co-infected individuals have lower intracerebral inflammation ^14^. A multicentre randomized controlled trial of adjunctive corticosteroids for HIV co-infected adults with TBM is currently underway in Vietnam and Indonesia ( NCT03092817) ^19^.

Corticosteroids are frequently used to treat common neuro-complications of TBM; paradoxical reactions, and the immune reconstitution inflammatory syndrome (IRIS). Paradoxical neuro-inflammatory reactions, which occur despite appropriate anti-TB chemotherapy, may reflect host response to dead and dying bacteria ^20^. TBM-IRIS is a common and often severe complication of starting antiretroviral therapy (ART) in TBM, and is associated with high CSF neutrophil counts and a positive *M.tb* culture at presentation ^21^. Interestingly, a CSF inflammatory process, specifically high neutrophils and high TNF-α in combination with low IFN-gamma, predicted later TBM-IRIS in a study of 34 individual with TBM in South Africa ^21^. Inflammasome activation appears to be involved in the development of TBM-IRIS, with MMP-9 a possible mediator of brain tissue damage ^11^. Whilst corticosteroids during the first four weeks after initiation of ART reduced TB-associated IRIS in HIV co-infected individuals in a trial in South Africa, individuals with TBM were excluded ^22^. There are no randomized trials of corticosteroid therapy for TBM-IRIS, nor for paradoxical neurological reactions in HIV uninfected individuals. Table 1 summarises the current evidence for dexamethasone use in TBM, as well as for other host-directed therapies.

**Table 1.** Summary of clinical studies investigating the efficacy of dexamethasone, aspirin and thalidomide in TBM.

Reference	Intervention (drug, dose, duration)	Study design	Population	Primary outcome	Key findings
Mai *^27^*	Aspirin 81 mg vs. 1000 mg vs. placebo for 60 days	RCT: double-blind, placebo controlled	Adults, non-HIV, Vietnam n = 120	Mortality or stroke	No difference in two-month mortality. Subgroup analysis showed reduction in infarcts and death with aspirin 81 mg (15%) and 1000 mg (11%) compared to placebo (34%); p = 0.06
Misra ^28^	Aspirin 150mg vs. placebo	RCT: placebo controlled	Adults n=118	Mortality or stroke	Decreased three-month mortality (21.7%) vs placebo (43.4%); odds ratio = 3.17, 95%CI 1.21 - 8.31. Aspirin resulted in absolute risk reduction of stroke in 19.1% and significant reduction in mortality compared to placebo (21.7% vs 43.4%, p=0.02).
Misra ^29^	Aspirin 150mg	Retrospective cohort	n=135	Mortality	Non-statistical reduction in deaths (25%) at three months compared to standard TB treatment (17%).
Schoeman ^30^	Aspirin 75mg or 100mg/kg	RCT	Children n=146		No improved neurological or cognitive outcomes or survival with aspirin.
Schoeman ^31^	Thalidomide 6mg/kg, 12mg/kg, or 24mg/kg	Dose escalating pilot study	Children n=15	Safety and tolerability	Reduced CSF TNF-α in children with stage 2 TBM.
Schoeman ^32^	Thalidomide 24mg/kg for one month	RCT: double-blind	Children n=47		Discontinued prematurely due to side effects and deaths in thalidomide arm.
Thwaites ^1^	Dexamethasone	RCT: double- blind, placebo controlled	Adults, HIV and non-HIV n=545	Mortality	Reduced risk of death through nine months (relative risk 0.69, p=0.01) with dexamethasone.
Simmons ^15^	Dexamethasone	RCT: double- blind, placebo controlled	Adults n=87		Dexamethasone did not significantly alter tested CSF cytokines (TNF-α, IL-1β, IL-6, IL-8, IL-10, IL-12) over time vs. placebo.

RCT, randomised clinical trial; IL, interleukin; TNF, tumor necrosis factor; TBM, tuberculous meningitis; TB, tuberculosis; CSF, cerebrospinal fluid.

In childhood TBM, benefit from corticosteroids has been demonstrated in a number of studies ^2, 23– 25^. Unlike in adults, improvement in disability, albeit moderate, is described ^3^. Dosage and duration, however, is debated and in randomized trials dosage has varied between 1mg/kg and 4mg/kg daily, for 3–4 weeks. One trial compared three dosage regimens; 2 mg/kg/day over four weeks vs 4 mg/k/day over one week and 2 mg/k/day for the next three weeks vs 4 mg/kg/day over four weeks ^26^. In each group the initial four weeks was followed by four weeks of tapering. There was no difference in mortality between groups; however, prolonged periods of higher dose prednisolone were associated with new onset optic neuropathy and hydrocephalus ^26^. These findings highlight the delicate balance between moderating host immunity and avoiding the occurrence of adverse events. Further studies are needed to identify ideal dosage regimen, as well as explore host variability in response to corticosteroids in childhood TBM.

### Aspirin

Cerebral infarction occurs in 25–71% of TBM cases ^27, 33^, and stroke was associated with a two-fold increase in mortality in a recent meta-analysis ^34^. The inflammatory state occurring in TBM contributes to the pathogenesis of stroke. A prospective study of 146 TBM patients demonstrated an acute phase inflammatory response with significantly elevated cytokines (e.g. IL-2, IL-4, IL-6, IL-1β, IFN-γ, TNF- α) in blood and CSF ^35^. A hypercoagulable state was reflected by elevated protein C, factor VII, plasminogen activator inhibitor-1 and anticardiolipin antibodies, as well as decreased protein S in a case series of 16 children. Bleeding times were also markedly shorter and platelet counts remained markedly raised in this subgroup ^36^. Hypercoagulability has been shown to occur in adults with pulmonary tuberculosis, and may also contribute to pathogenesis of stroke in TBM. Local intra- and extra-vascular factors contributes to TBM pathogenesis, most significantly in the form of cerebral vasculitis secondary to inflammatory infiltrates; initially believed to be directly due to tubercle bacilli implantation, now known to correlate with the inflammatory exudate in the basal cisterns and subarachnoid space ^37, 38^. The significance of intravascular thrombosis is still unclear. While thrombosis might be common in the context of vasculitis, autopsies on TBM patients failed to demonstrate frequent arterial thrombosis ^37^. Significant platelet dysfunction has also been demonstrated in TBM, manifesting as increased mean platelet volumes, platelet distribution width and platelet-large cell ratio ^39^. These parameters are significantly associated with infarcts and suggests the use of antiplatelet agents in TBM ^39^. Local intra- and extra-vascular factors contribute to TBM pathogenesis in the form of vasculitis due to bacilli infiltration. In an effort to reduce mortality and long-term neurological disability in TBM, aspirin is increasingly being studied due to its anti-inflammatory and inhibitory effects on platelet and thrombus production. In murine models, low dose aspirin (3 mg/kg) showed a systemic decrease in serum cytokines (e.g. TNF-α, IL-6, IL-1β) and late stage T cell responses in *M.tb* infection. Aspirin also enhances T helper cell 1 responses for eliminating bacilli from lungs ^40^.

To date, three randomized controlled trials have investigated the role of aspirin in adult and paediatric TBM. In 118 adult TBM patients in India, aspirin resulted in absolute risk reduction of stroke in 19.1% and significant reduction in mortality compared to placebo (10 of 118 (21.7%) versus 23 of 118 (43.4%), p=0.02) ^28^. A randomised controlled trial of TBM involving children in South Africa (n=146) could not establish improved neurological/cognitive outcomes or survival with aspirin at doses of 75 mg (low dose) or 100 mg/kg/day (high dose) ^30^. However, the developmental outcome of children on high dose aspirin was similar to the placebo and low dose aspirin groups, despite being younger of age and having higher baseline severity. This finding warrants further investigation of high-dose aspirin in childhood TBM. A study of 120 Vietnamese adults with TBM demonstrated a reduction in death and new infarcts with the addition of 81 mg/day aspirin (8 of 36 or 22.2%) and 1000 mg/day aspirin (6 of 38 or 15.8%), versus placebo (11 of 38 or 28.9%) ^27^. Aspirin was associated with dose-dependent inhibition of thromboxane A2 and upregulation of pro-resolving protectins in the CSF. Another retrospective study by Misra *et al.* in India failed to validate clinical benefit, showing an insignificant reduction in deaths with the addition of 150 mg aspirin as compared to standard anti-TB therapy ^29^. However, 25% (11 of 135) of patients randomized to the aspirin arm had a complete recovery at three months versus 17.1% (7 of 135) in the standard treatment arm. In the three adult trials, corticosteroids were administered alone or in conjunction with aspirin with no adverse event signal found. None of these trials observed an increase in adverse events, but safety concerns with increasing doses of aspirin persist. Whilst these studies of adjunctive aspirin described varying results regarding morbidity and mortality, they paved the way for further large randomised controlled trials. Phase 2 ( NCT03927313) and 3 ( NCT04145258) trials are currently underway to validate aspirin as a host-directed therapy. Given the insufficient evidence base, aspirin is not routinely used in most individuals with TBM.

### Thalidomide

Thalidomide has a wide range of biological effects, due to its ability to interfere with the immune system, and depending on the cell type or pathway of activation. The inhibition of TNF-α, which is produced primarily by macrophages and monocytes, accounts for most of the immunological effects of the drug. TNF-α performs a delicate balancing act during host response to *M.tb* infection, whereby on the one hand it is mandatory for keeping infection under control, but on the other hand, if produced at too high levels it induces a hyperinflammatory state resulting in severe tissue damage. The potential of thalidomide to activate T-cells, resulting in elevated production of IL2, IFN and TNF-α, may potentially interfere with its anti-inflammatory properties ^41^. In addition, thalidomide does not inhibit TNF-α produced by stimulated T-cells. The therapeutic effect of thalidomide therefore appears to be dose dependant since differing TNF-α concentrations will result in opposing physiological consequences. 

Thalidomide has been shown to reduce CSF TNF-α experimentally in rabbits ^42^ as well as in children with UK Medical Research Council (MRC) grade 2 TBM in a dose-escalating pilot study ^31^. However, a double-blind, randomized trial of high dose thalidomide treatment (24mg/kg/day for one month) in children with grade 2 and 3 TBM was discontinued due to side effects (skin rash, hepatitis, neutropenia or thrombocytopenia) and deaths in the thalidomide arm ^32^.

**Figure 1.**
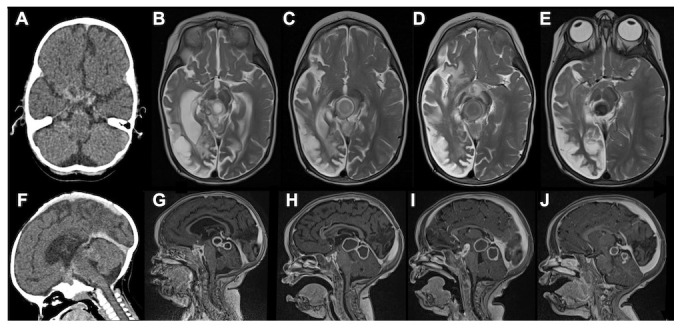
CT axial, MRI T2 axial, CT sagittal and MRI T1 post-gadolinium sagittal images at 3–4 month intervals of a 16-month-old HIV-infected female with stage III tuberculous meningitis. The initial computed tomography (CT) axial and sagittal scans ( **A**, **F**) showed a large right sided middle cerebral artery infarction, hydrocephalus as well as multiple small rim-enhancing foci in the prepontine cisterns. After three months of anti-TB and two months of anti-retroviral therapy, they presented with a depressed level of consciousness. Magnetic resonance imaging (MRI) T2 axial ( **B**) and MRI T1 post-gadolinium sagittal ( **G**) demonstrated multiple TB abscesses in the interpeduncular, prepontine and chiasmic cisterns (paradoxical HIV related TB IRIS) as well as right cerebral hemisphere spongiotic changes (old infarction). Thalidomide was initiated following a poor response to one week of high dose corticosteroids. This resulted in rapid improvement in the level of consciousness, gradual decrease in the size of the TB abscesses and loss of T2 signal (i.e. inflammation), which is a marker of cure as it represents gradual calcification ( **C**– **E** & **H**– **J**). We confirm that we have obtained consent to use images from the parent/guardian of the patient included in this presentation. Permission was obtained in the form of informed written consent.

The anti-inflammatory benefits of thalidomide (e.g. improved resolution of basal enhancement and tuberculomas) noted in both the pilot and randomized trials have led to more targeted studies, albeit at a much reduced dosage (≤5 mg/kg/day). Additionally, adjunctive thalidomide has been shown to be particularly effective in observational studies involving tuberculous brain abscesses ^43, 44^ and blindness-related to optochiasmatic arachnoiditis ^45, 46^. Adverse drug effects have been less of an issue in these situations. The life-threatening nature of these TBM sequelae as well as the anatomical location of the lesions, which precluded surgery, disqualified them from being included in trials. Nonetheless, the clinical improvements noted have been substantial ( Figure 1).

When used, the duration of adjunctive thalidomide therapy should be guided by subsequent clinical and radiological responses. In TB, clinical improvement of mass lesions generally precedes radiological improvement due to a reduction in peri-lesional inflammation. Serial MRI T2-weighted studies have shown that evolution of the lesions from early stage “T2 bright” abscesses with oedema to “T2 black” represents a marker of cure ^44^. Regression is associated with fibrosis, mineralization (calcification) and eventually disappearance, usually with no residual structural abnormalities. T2-black granulomas may, however, persist for years in asymptomatic children. In most cases, cure is achieved after less than three months of adjunctive thalidomide therapy.

It is the authors experience that adjunctive thalidomide warrants consideration in the following TBM-related conditions: corticosteroid-unresponsive optochiasmatic arachnoiditis resulting in visual impairment and/or optic disc pallor; enlarging TB abscess despite corticosteroid therapy (TB-IRIS); large TB abscess/tuberculomas in critical brain regions (i.e. brainstem) that is not amenable to surgical drainage and not responding to corticosteroids; large dural-based TB abscess resulting in epilepsia partialis.

TNF-α has been shown to exert deleterious effects on capillaries already sensitized by exposure to mycobacterial products. The endarteritis, coupled with raised intracranial pressure because of edema and obstructive hydrocephalus, often leads to cerebral ischaemia/infarction. The value of low-dose adjunctive thalidomide in modifying the progressive endarteritis is yet to be explored.

### Immunomodulatory therapies

Modulation of cytokines known to contribute to pathology is a potential strategy to support host defenses or control deleterious inflammation in TBM. In TBM, a number of pro-inflammatory cytokines are thought to play a role in pathogenesis, including IL-2, IL-6, IL-1β, IFN-γ and TNF-α ^38^. However, like in other neuroinflammatory conditions where cytokines such as IFN-γ have opposing roles ^47^, inhibition of these cytokines may not necessarily lead to improved outcomes and therefore caution must be exercised in exploring the potential drugs that inhibit these pro-inflammatory cytokines as candidate HDTs.

There are accumulating data on the role of the anti-TNF-α monoclonal antibodies infliximab and adalimumab and the soluble TNF-α receptor etanercept in TBM treatment. Although these agents are described as options for treating refractory paradoxical reactions involving the CNS ^48– 51^, they may also be responsible for latent TB reactivation and dissemination to the CNS in those where the drug is used to treat autoimmune conditions ^52^. Anakinra is a human interleukin-1 receptor antagonist that blocks the biological activity of natural IL-1 and may also have a role in TBM. Anakinra demonstrated efficacy in one case of life-threatening protracted paradoxical inflammation in CNS TB where high dose corticosteroids failed ^53^. Other immunomodulatory agents of interest include canakinumab and tocilizumab, human monoclonal antibodies inhibiting IL-1 and IL-6, respectively. In TBM, vasculitis occurs due to the proximity of the progressive exudative meningitis to the basal subarachnoid cistern and the circle of Willis. Cyclophosphamide, an alkylating cytotoxic drug is an effective drug in the treatment of primary cerebral vasculitis. Two case reports have described clinical improvement with the use of cyclophosphamide in TBM associated cerebral vasculitis ^54, 55^; however, its role as an effective treatment in this context needs further investigation, particularly due to concerns over its potential adverse activity as a potent immunosuppressive drug. Table 2 summarises cases within the published literature where these agents have been used in the context of TBM; however, pre-clinical and clinical studies to systematically investigate the therapeutic effectiveness are required before they can be used more widely as adjunctive therapies in TBM.

**Table 2.** Biologics and other immunomodulatory therapies in TBM; summary of published case reports.

Reference	Drug	Dose	Mechanism	Clinical outcome
Blackmore ^48^	Infliximab	10mg/kg, three doses at monthly intervals	Anti-TNF	Given after four months due to ongoing clinical deterioration, despite treatment with dexamethasone and cyclophosphamide; resulted in clinical improvement.
Jorge ^49^	Infliximab	10mg/kg, three doses at monthly intervals	Anti-TNF	Young adult with juvenile idiopathic arthritis, treated with infliximab developed disseminated TB. With stopping of infliximab, neurological deterioration occurred with isolation of *M.tb* in CSF, with no improvement with corticosteroids. Infliximab re- initiation led to neurological improvement.
Molten ^50^	Infliximab	Case 1: 10mg/kg, three doses at monthly intervals Case 2: 5mg/kg, three doses at six- week intervals	Anti-TNF	Two cases describing paradoxical worsening after initiation of TBM treatment, unresponsive to dexamethasone. In both cases, clinical improvement occurred following administration of infliximab.
Abo ^51^	Infliximab	5mg/kg, three doses at weeks 1, 3 and 7	Anti-TNF	Paradoxical worsening (optochiasmatic arachnoiditis, leading to loss of vision) on starting TB treatment in a seven-year-old with TBM, despite dexamethasone. Clinical improvement occurred following infliximab administration.
Keeley ^53^	Anakinra	100 mg subcutaneously daily	Interleukin-1 receptor antagonist	Two cases of steroid dependant neurotuberculosis (paradoxical worsening when steroids stopped). In both cases, patients responded to anakinra therapy.
A. Gonzalez- Duarte ^55^	Cyclophosphamide	750mg/m ^3^ every three weeks	Alkylating agent of nitrogen mustard type.2	Clinical improvement
Celloti ^54^	Cyclophosphamide	750mg/m ^3^ every three weeks	Alkylating agent of nitrogen mustard type.2	Clinical improvement

TNF, tumor necrosis factor; TB, tuberculosis; *M.tb*, *Mycobacterium tuberculosis*; CSF, cerebrospinal fluid; TBM, tuberculous meningitis.

### Antiretroviral therapy

The decision as to when antiretroviral therapy is started must consider the potential immunopathogenic complications as well as the benefit in preventing further opportunistic infection. Guidelines vary slightly regarding the timing of initiation of ART relative to initiation of anti-TB chemotherapy in those co-infected with TB and HIV. The 2010 World Health Organisation (WHO) ART guidelines recommend initiating ART within eight weeks of anti-TB chemotherapy in all HIV-TB co-infected patients regardless of CD4 count ^56^. The U.S. National Institutes of Health HIV guidelines recommend starting ART within two weeks of anti-TB chemotherapy for HIV-TB co-infected patients with CD4 cell counts <50 cells/ µL and within eight weeks for CD4 counts >50 cells/µL ^57^. In TBM there are unique considerations given the infection surrounds crucial structures (the brain and spinal cord) with a very limited ability to expand within the skull and spinal canal should excess inflammation occur. Inflammation occurring following the initiation of HIV therapy is known as immune reconstitution inflammatory syndrome (IRIS), which in the context of TBM is associated is frequent (up to 40%) and associated with high mortality (30%) ^58, 59^.

In a randomised trial of testing immediate HIV therapy initiation (at time of initiating TB treatment) vs delayed (after two months) in TBM, immediate therapy was associated with significantly more grade 4 adverse events (n=102) than delayed HIV therapy (n=87; p=.04). This trial informed current consensus that ART initiation should be delayed by between 4-8 weeks after starting TBM therapy ^60^. This approach hopes to strike a balance between the beneficial effects of ART (immune reconstitution, control of HIV, prevention of other opportunistic infections) and the potential harms of TB-IRIS.

## Potential future host directed therapies for tuberculous meningitis

**Figure 2.**
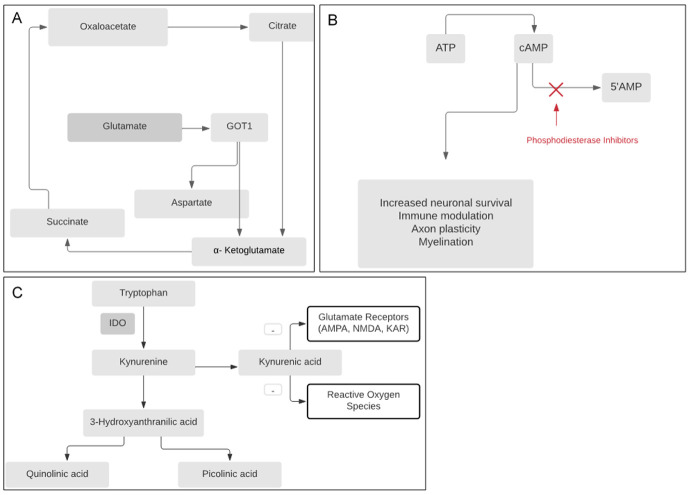
Schematic of relevant biochemical pathways implicated in development of emerging host directed therapies. **A:** Drugs that reduce glutamate ‘glutamate grabbers’ by increasing breakdown of glutamate (either recombinant glutamic-oxaloacetic transaminase (GOT1), or others that mimic its action) may decrease neuro-excitotoxicity associated with brain injury in TBM. **B:** Phosphodiesterase inhibitors reduce breakdown of cAMP to 5’AMP, leading to increased neuronal survival, immune modulation, and increase in axon plasticity and myelination. **C:** Interruption of the tryptophan pathway via modulation of indoleamine 2,3-dioxygenase (IDO) activity may have neuroprotective effect, although more data to understand the role of downstream metabolites, particularly the contribution of kyruneic acid in antagonism of glutamate receptors, is needed.

Although host directed therapies are in use, they are limited in either efficacy or availability. Therefore, the quest for more effective therapeutics remains ongoing. Here we discuss potential therapies that target pathways highlighted in recent pathogenesis studies, or draw on insights from other forms of TB or inflammatory conditions with shared mechanisms of pathogenesis ( Figure 2).

### Statin therapy

HMG-CoA reductase inhibitors (‘statins’) are ubiquitously used in prevention and treatment of cardiovascular disease, but are also known to have immunomodulatory, anti-inflammatory and anti-oxidative properties. Several *in vitro* studies have demonstrated that statins enhance anti-inflammatory and inhibit pro-inflammatory functions in microglial cells and inhibit mechanisms involved in neurodegeneration ^61– 64^. Anti-inflammatory properties may be due to modulation of isoprenylation ^65^ with downstream effects on inhibitory and stimulatory transcription pathways, or via allosteric inhibition of leucocyte function antigen (LFA)-1 integrin ^66^, which is involved in the transmigration of activated T cells through the blood brain barrier. Neuroprotective effects may be due to modulation of excitotoxicity, vascular function, angiogenesis, and/or reduced oxidative damage through nitric oxide stimulas ^67, 68^. Importantly, some studies have shown increased neuronal death with higher concentrations of statins ^69– 71^.

The potential of statins to effect CNS inflammation and neurodegeneration in other conditions are of interest given the shared mechanistic pathways in TBM. For example, animal models of multiple sclerosis (MS) show that statins skew immune responses towards an anti-inflammatory T-helper cell 2 response, inhibiting pro-inflammatory cytokines IL-2, IL-12 and IFN-γ ^72^. Patients with secondary progressive MS benefited from statin therapy ^73^ and a phase 3 trial is currently underway to examine this further ( NCT03387670). In a mouse model of traumatic brain injury, atorvastatin led to profound attenuation of T cell, neutrophil and natural killer cell invasion into the CNS, and reduction in production of pro-inflammatory cytokines (IFN-y and IL-6) and chemokines (CCL5 and CXCL10) ^74^. In a retrospective observational study, pre-injury statin therapy was associated with improved neurological outcomes in mild traumatic brain injury ^75^; however, in moderate to severe injury no benefit was noted ^76^. In a double-blind randomised trial involving 36 patients with traumatic brain injury, rosuvastatin given for 10 days in the acute phase of injury significantly reduced TNF-α, which correlated with a reduction in disability scores ^77^. Other conditions where the role of statins has been explored include Alzheimer’s disease ^78^, and Parkinson’s disease ^79^. Further, statins may be associated with reduced risk of tuberculosis ^80^. In a TB murine model, adjunctive simvastatin shortened time to culture clearance by 1 month, enhanced bacterial killing, and decreased culture-positive relapse and enhance bacterial killing ^81– 83^. Clinical trials ( NCT03456102, NCT04147286) will investigate the efficacy of statins in pulmonary tuberculosis. Given their potential use as an adjunctive TB therapy, their lipophilic properties allowing good penetration to the CNS, as well as their potential as an anti-inflammatory and neuroprotective agent, statins may have a role as a HDT in TBM; trials to explore this hypothesis are needed.

### Glutamate ‘grabbing’ drugs

Excessive glutamate and neuro-excitotoxicity are thought to contribute to brain injury and cell death in TBM. In one study, RNA sequencing of whole blood and CSF from children with TBM demonstrated significant enrichment of transcripts associated with neural excitotoxicity predominantly driven by glutamate release, NMDA receptor binding and uptake ^84^. This mechanism is thought to contribute to brain injury and cell death in other neurological conditions such as stroke, epilepsy, traumatic brain injury, Alzheimer’s and Huntington’s disease ^85, 86^. Therapeutics that aim to reduce glutamate excitotoxicity either by i) modulating the downstream effects of glutamate via NMDA receptor binding or ii) reducing extracellular glutamate (e.g. glutamate ‘grabbing’) may have a role in the treatment of TBM. In acute stroke, a similar approach was taken; however, although animal studies were promising, randomised trials in humans assessing efficacy of NMDA antagonists largely failed ^87– 89^. Therapeutics have been designed to reduce glutamate induced excitotoxicity by lowering blood glutamate concentration, thus leading to a larger natural glutamate gradient between the brain and blood, thereby facilitating the efflux of extracellular brain glutamate into the blood ^90^. In an animal study, riboflavin (vitamin B _2_), selected for its ability to interact with glutamate-oxaloacetate transaminase (GOT), significantly reduced blood glutamate levels compared to placebo ( Figure 2A) ^91^. In a randomised trial, riboflavin was correlated with improvement of disability when given intravenously in adults with acute stroke ^91^. A number of studies have explored the neuroprotective properties of riboflavin including in conditions such as migraine and Parkinson’s disease ^92^. It is unclear whether drugs such as riboflavin, or others that reduce glutamate neuro-excitotoxicity, have a role as an adjunctive therapy to promote neuroprotection in TBM; however, given the emerging body of evidence that suggests involvement of the glutamate-glutamine pathway, this is a potential area of interest for future studies.

### Tryptophan pathway drug targets

Tryptophan is an essential amino acid that can either be converted to serotonin or oxidized kynurenines via indoleamine 2,3-dioxygenase (IDO1) ( Figure 2C). Further oxidization occurs to convert kynurenine to kynurenic acid, which has neuroprotective properties. Prior studies have shown that *M.tb* induces marked upregulation of IDO-1 expression in both human and murine macrophages *in vitro* ^93^, and that blockade of IDO activity reduces both clinical manifestations of TB as well as microbial and pathological correlates of the human TB syndrome in macaques ^94^. In an observational cohort study of TBM, *low* CSF tryptophan levels were found in those who survived, compared to non-survivors or controls ^95^. It is therefore unclear in TBM whether drugs that block IDO-1 such as indoximod, an immunometabolic adjuvant that is current under investigation in cancer therapy ^96^, would cause benefit or harm. It is plausible that improved survival seen in those with low CSF tryptophan is due to increased availability of kynurenic acid, which has neuroprotective action via glutamate receptors and reactive oxygen species. Further investigation into the influence of tryptophan and its downstream metabolites on pathogenesis in TBM is required in order to establish suitable targets along this pathway for HDTs.

### Eicosanoid modulating drugs

Eicosanoids are arachidonic acid derived lipid mediators that trigger pro-and anti-inflammatory responses and include prostaglandins, resolvins, lipoxins, and leukotrienes, which serve as signalling molecules, modulating inflammation and cell death in TB ^97^. A delicate balance in eicosanoid levels is crucial for *M.tb* control and regulating the production of pro-inflammatory cytokines ^98^.

Non-steroidal inflammatory drugs (NSAIDs), which exert their effects by inhibiting cyclooxygenase (COX) activity may lead to reduction of excessive inflammation in TBM. As discussed, aspirin, a non-selective COX inhibitor, has been investigated in three trials in TBM with variable outcomes ^27, 28, 30^. New generation NSAIDs with more selective inhibition of COX2 may have more favourable safety profiles. Phase 1 trials to assess the safety and bactericidal activity of celecoxib and etoricoxib in healthy volunteers with a view to developing these agents as HDTs for drug sensitive TB are currently underway ( NCT02602509, NCT02503839). Although trials to further investigate the role of aspirin in TBM are underway, future research should consider the potential contribution of newer more selective COX2 inhibitors in TBM.

### Phosphodiesterase inhibitors

Phosphodiesterase inhibitors (PDE-i) are small-molecule inhibitors that reduce inflammation by increasing intracellular cyclic adenosine monophosphate and cyclic guanine monophosphate ^99^ ( Figure 2B). Phosphodiesterase 4 (PDE-4) inhibitors such as roflumilast have shown to be effective in the treatment of numerous inflammatory conditions including chronic obstructive inflammatory disease ^100^. PDE-4 is expressed within the cortex and hippocampus and animal models suggest that inhibition of PDE-4 may have a beneficial role in CNS conditions where inflammation plays a role in pathogenesis ^101– 105^. In animal models of pulmonary TB, inhibition of PDE-3 (cilostazol), PDE-4 (roflumilast) and PDE-5 (sildenafil) have all increased bacterial clearance and reduced pro-inflammatory cytokines, which contributed to a reduction in neutrophil infiltration and lung pathology ^106– 109^. The role of phosphodiesterase inhibitors has not been studied in TBM but the properties above make them intriguing candidates for adjunctive therapy in TBM. 

## Variable host responses and a personalized approach

Host immune response to *M.tb* in TBM is vital, although excessive inflammation leads to neurological damage. Polymorphisms in genes involved in immune response or signalling pathways can influence host inflammatory response, or susceptibility to TBM ^110^.

Previous study in the zebrafish model showed that the leukotriene A4 hydrolase ( *LTA4H*) gene influenced the balance of pro and anti-inflammatory eicosanoids in response to *M.tb* infection ^111^. LTA4H catalyses the final step in pro-inflammatory leukotriene B4 (LTB4) synthesis ^111^, with LTB4 effects usually balanced by anti-inflammatory lipoxin A4 (LXA4), the two together ensuring an appropriate response to *M.tb* without excessive tissue damage ^112^. A single nucleotide polymorphism (SNP) (rs17525495) in the promoter region of the *LAT4H* gene alters gene expression, and LTB4 LXA4 balance; low (CC) and high (TT) inflammatory states result from *LTA4H* allele homozygosity whereas an intermediate (CT) inflammatory state results from allele heterozygosity ^111^. Both TT and CC inflammatory states were associated with increased death in a retrospective study of adults with TBM ^113^. In this retrospective study adjunctive dexamethasone was associated with improved survival in the high inflammatory TT group, with the effect of dexamethasone unclear in the CC and CT groups ^113^. In a subsequent study of 764 Vietnamese adults, 10 CSF cytokines were measured of: TNF-α, IFN-γ, IL-1β, IL-2, IL4, IL-5, IL-6, IL-10, IL-12, IL-13 ^14^. In HIV-uninfected adults with TBM, pro-inflammatory IL-1β, IL-2, and IL-6 (but not TNF-α) were significantly associated with *LTA4H* genotype; low concentrations in CC genotype, intermediate concentrations in CT genotype, and high concentrations in TT genotype ^14^. In HIV co-infected individuals with TBM, *LTA4H* genotype did not appear to influence survival, response to dexamethasone, or CSF cytokine profile ^14^. Additionally, *LTA4H* genotype did not influence survival in a study of HIV-uninfected Indonesian adults with TBM, all of whom received corticosteroids ^114^. A *LTA4H* genotype stratified approach to adjunctive corticosteroid therapy in TBM is now being assessed in an ongoing randomized placebo-controlled *LTA4H* genotype stratified non-inferiority trial of HIV uninfected adults with TBM in Vietnam ( NCT03100786) ^115^. If benefit of adjunctive corticosteroids as a host directed therapy are shown to be limited to one or more *LTA4H* genotypes, this paves the way for personalized corticosteroid therapy in TBM.

Where variable host responses to *M.tb* increase intracerebral inflammation, or genetic polymorphisms lead to overexpression of a specific molecule or target, targeted personalized therapies may be beneficial. In a study of tryptophan genome wide SNP data we identified 11 quantitative trait loci associated with CSF tryptophan concentrations, and found that these quantitative trait loci were predictive of patient survival ^19^. A SNP (rs17842268) in CD43, a surface glycoprotein, has been associated with more severe presentation, and decreased survival, in TBM ^116^. Why SNPs in CD43 affect *M.tb* susceptibility is uncertain, but CD43 has a role in regulating proinflammatory cytokines ^116^, and theoretically anti-inflammatory therapies may be beneficial in such patients. Evidence that patients with a dysregulated host immune response benefit from more, or different, host directed therapies is lacking.

## Conclusions and key areas for future research

Host directed therapies are an evolving area of TBM research. We know that the inflammatory response in TBM contributes to poor outcomes. Further, we know that dexamethasone reduces death from TBM. What is unknown is how the drug works, who might benefit most from dexamethasone or whether other therapies should be given in addition to dexamethasone or in place of it in some scenarios. There may also be scenarios where dexamethasone is harmful. Important questions regarding the exact role of thalidomide and aspirin also remain. While in the case of the former, a narrow context in which the drug might be useful is becoming clearer, in the latter, the optimal and safe dose of aspirin, considering its antiplatelet and anti-inflammatory properties, is uncertain. Although the use of immunomodulatory therapies has been reported sporadically, often where corticosteroid treatments have failed, no clinical trials have been conducted to systematically assess their safety profile and efficacy.

Drug discovery depends on accurately identifying molecular targets that play crucial roles in disease biology, and which are amenable to modulation via biologics or small molecule drug therapeutics. In diseases with high global incidences such diabetes or hypertension, large scale data repositories are beginning to provide genetic insights to inform drug discovery and therefore change the direction of and speed at which novel and repurposed therapeutics become available ^117^. In TBM we must work towards establishing similar repositories through international collaboration. However, the relatively low global incidence of the disease, and the challenging environments in which TBM most commonly occurs will make this a lengthy endeavour.

In the near future, we can focus on better understanding of key pathogenic processes underpinning inflammation and brain injury. For example, further understanding of the role and interaction of glutamate and tryptophan in brain injury may uncover targets for which existing drugs can be repurposed and novel therapeutics developed. The rational design of animal models to help inform which of these might deserve clinical trials in TBM is also key; although the rabbit model of TBM has been in use since the early 1900s ^118^, further research is required to establish whether a more refined or alternative model could better recapitulate human disease. Genomic research to identify variation in host response will allow further refinement of therapeutic approaches based on factors at the individual patient and population level. While studies of LTA4H genotype have led the way in this area of TBM research, focus must now widen to include other pathways that are likely to vary between hosts. As we move forward with host-directed therapies for TBM, we must remain cognisant of the characteristics of the hosts whose responses we are attempting to change. Whether these changes are obvious (e.g. HIV infection) or more opaque (e.g. unknown genetic polymorphisms), they must be considered with trial design so that we can understand as fully as possible, the role of these therapies in improving outcomes in TBM.

## Data availability

No data are associated with this article.
