# Supplementary material for: Host Directed Therapies for Tuberculous Meningitis
Source: Wellcome Open Res. 2021 Jul 1;5:292. Originally published 2020 Dec 23. [Version 2] doi: 10.12688/wellcomeopenres.16474.2 (PMC8792876; doi:10.12688/wellcomeopenres.16474.2)
Supplement: Peer review history

# Peer review: Reviewer response for version 2

**DOI:** 10.21956/wellcomeopenres.18684.r44724

**Published:** 2022-01-26

**Authors:** Fabrice Bonnet

**Affiliations:** 1 ISPED, Centre INSERM U1219, Bordeaux Population Health Research Center, University of Bordeaux, Bordeaux, France

I have no additional comments for this submission.

Is the review written in accessible language?

Yes

Are all factual statements correct and adequately supported by citations?

Partly

Are the conclusions drawn appropriate in the context of the current research literature?

Yes

Is the topic of the review discussed comprehensively in the context of the current literature?

Yes

Reviewer Expertise:

NA

I confirm that I have read this submission and believe that I have an appropriate level of expertise to confirm that it is of an acceptable scientific standard.

# Peer review: Reviewer response for version 1

**DOI:** 10.21956/wellcomeopenres.18139.r42998

**Published:** 2021-04-12

**Authors:** Vishwanath Venketaraman

**Affiliations:** 1 College of Osteopathic Medicine of the Pacific, Western University of Health Sciences, Pomona, CA, USA

This review is well-written with complete focus on the potential host directed therapies for TB meningitis. The manuscript contains a detailed literature review (both preclinical and clinical trial studies) on the use of corticosteroids, aspirin, thalidomide, statins, and other molecules, against TB meningitis. 

 Recommendations: Introducing a paragraph about tuberculosis incidence and epidemiology at the beginning of the manuscript, followed by sections on the prevalence of TB meningitis, and then pathogenesis of TB meningitis, will further enhance the strength of the manuscript.

 The reviewer has provided specific comments within the text of the manuscript.

Is the review written in accessible language?

Yes

Are all factual statements correct and adequately supported by citations?

Yes

Are the conclusions drawn appropriate in the context of the current research literature?

Yes

Is the topic of the review discussed comprehensively in the context of the current literature?

Yes

Reviewer Expertise:

Tuberculosis, HIV and Diabetes

I confirm that I have read this submission and believe that I have an appropriate level of expertise to confirm that it is of an acceptable scientific standard.

# Response

**Published:** 2021-05-19

**Authors:** Angharad Davis

**Affiliations:** University College London, UK

Dear Reviewer 2, 

 Many thanks for your thorough review of the manuscript and your suggestion to add a section on TBM epidemiology and pathogenesis.

 We agree that these are important areas, however since this manuscript forms a collection of published papers in a TBM supplement, many of which covered TBM epidemiology and pathogenesis in detail, the authors felt that this manuscript should maintain a narrow focus on the subject area, and therefore felt chapters on TBM epidemiology/pathogenesis was beyond the scope of the article.

# Peer review: Reviewer response for version 1

**DOI:** 10.21956/wellcomeopenres.18139.r41926

**Published:** 2021-02-05

**Authors:** Fabrice Bonnet

**Affiliations:** 1 ISPED, Centre INSERM U1219, Bordeaux Population Health Research Center, University of Bordeaux, Bordeaux, France

**General comments:**

 This review regarding the host directed therapy (HDT) for TBM is globally well written and includes adequate references on this topic to date. It could be better balanced.

 In the introduction, the authors could include a short chapter about TBM epidemiology.

**Systematic review:**

**Title**

 I do not agree to consider aspirin, thalidomide and immunomodulatory therapies (anti-TNF and anti-Il1 antibodies) as “existing “ HDT in TBM. To date, only one HDT has been proven to reduce mortality, namely dexamethasone and the other one are promising approaches but have not been assessed in clinical trials yet. So I would consider as Existing HDT the only dexamethasone and “Promising” HDT the other ones.

 The title “potential future HDT…” should be replaced by Potential “pathways” for HDT since some of the activators/inhibitors cited in this chapter are new components never evaluated in RCTs.

**Chapter Dexamethasone:**

 The authors should describe what kind of controls were evaluated in a study of 16 individuals in India.

 The authors write about cerebral inflammation but it is a proxy to consider CSF cytokines as a maker of cerebral inflammation. The authors should comment on this point.

 The issue of dexamethasone dosage (and of rifampicin induction) and of the use of other corticosteroids should be detailed in this chapter described since dexa is the only HDT validated in the guidelines.

**Chapter Aspirin:**

 The chapter is well written.

 In addition to the comprehensive antiagregant effect of aspirin, can the author explain how they expect an additional antiinflammatory effect to high dose dexamethasone?  The duration of the treatment should be discussed.

**Chapter Thalidomide:**

 Data on thalidomide are very few and mainly observed in children.

 The authors should be cautious regarding their experience with adjunctive thalidomide in TBM regarding the lack of date. Thalidomide is an old anti-inflammatory drug used in inflammatory diseases and progressively removed from guidelines because of its toxicity. Again, it seems difficult to consider thalidomide as a “promising” drug for TBM since new biological agents are far more efficient on proinflammatory cytokines.

 The authors should not suggest using thalidomide at a large scale in TBM/TB brain abscess.

**Chapter Immunomodulatory HDT:**

 This chapter is quite short regarding the perspective given by anti-TNF in a severe form of TBM/CNS TB. The choice of the type of anti-TNF, as well as the duration of treatment and the risk associated to this medication, could be discussed. To my mind, anti-TNF therapies may represent one of the best approaches to reduce mortality and morbidity of TBM/TB Abssess in HIV and non HIV people and in children. The main issues are the toxicity of these drugs in the environment where TBM occurs and the availability of these drugs in poor income countries. These points have to be discussed in this review.

 In table 2, the authors should add the following references about adalimumab:

Adalimumab treatment may replace or enhance the activity of steroids in steroid-refractory tuberculous meningitis.  Lee HS, Lee Y, Lee SO, Choi SH, Kim YS, Woo JH, Kim SH. J Infect Chemother. 2012 Aug;18(4):555-7

Adalimumab for Corticosteroid and Infliximab-Resistant Immune Reconstitution Inflammatory Syndrome in the Setting of TB/HIV Coinfection.  Lwin N, Boyle M, Davis JS. Open Forum Infect Dis. 2018 Jan 30;5(2):ofy027

**ART:**

 It is not really fair to consider ART as HDT… even if it is obvious that ART has a significant impact on immunological status. HIV coinfection raises other issues in the management of TBM and I would consider separating this chapter from HDT.

**Chapter Future pathways…:**

 Chapter Statin therapy

 It is not well balanced to have a chapter statin with no data on TBM longer than the chapter on biological agents, even if the background is interesting.

 I would summarize the impact of statin on multiple sclerosis,  brain injury, etc since it is quite far from TBM to focus on the potential interest of statins on TB and immune response.

 FIGURE 2: Not really helpful in the present form.

 I would perform a central plot with increased neuronal survival, immune modulation, axon plasticity myelination that are the goal of treatment; and to describe around the central plot the different pathways to reach this target.  

**Chapter Host response:**

 The discussion regarding the LTA4H gene and the response to dexamethasone is an important point of pharmacogenetic. The authors should discuss if this strategy is affordable in every setting.

Is the review written in accessible language?

Yes

Are all factual statements correct and adequately supported by citations?

Partly

Are the conclusions drawn appropriate in the context of the current research literature?

Yes

Is the topic of the review discussed comprehensively in the context of the current literature?

Yes

Reviewer Expertise:

HIV Infection ; TBM

I confirm that I have read this submission and believe that I have an appropriate level of expertise to confirm that it is of an acceptable scientific standard, however I have significant reservations, as outlined above.

# Response

**Published:** 2021-05-19

**Authors:** Angharad Davis

**Affiliations:** University College London, UK

Dear reviewer 1,

 Many thanks for your thorough review of the manuscript and your suggestions. We have addressed each in turn in the responses below.

**General comments:**

 This review regarding the host directed therapy (HDT) for TBM is globally well written and includes adequate references on this topic to date. It could be better balanced.

 In the introduction, the authors could include a short chapter about TBM epidemiology.

 We agree that this is an important area, however since this manuscript forms a collection of published papers in a TBM supplement, many of which covered TBM epidemiology in detail, the authors felt that this manuscript should maintain a narrow focus on the subject area, and therefore felt a chapter on TBM epidemiology was beyond the scope of the article.

**Paragraph sub-headings:**

 I do not agree to consider aspirin, thalidomide and immunomodulatory therapies (anti-TNF and anti-Il1 antibodies) as “existing “ HDT in TBM. To date, only one HDT has been proven to reduce mortality, namely dexamethasone and the other one are promising approaches but have not been assessed in clinical trials yet. So I would consider as Existing HDT the only dexamethasone and “Promising” HDT the other ones.

 The title “potential future HDT…” should be replaced by Potential “pathways” for HDT since some of the activators/inhibitors cited in this chapter are new components never evaluated in RCTs.

 We have changed the sub-headings as suggested.

**Chapter Dexamethasone:**

 The authors should describe what kind of controls were evaluated in a study of 16 individuals in India.

 This has been added to this section.

 The authors write about cerebral inflammation but it is a proxy to consider CSF cytokines as a maker of cerebral inflammation. The authors should comment on this point.

 A comment has been added to this section as per suggestion .

 The issue of dexamethasone dosage (and of rifampicin induction) and of the use of other corticosteroids should be detailed in this chapter described since dexa is the only HDT validated in the guidelines.

 We agree this issue is important and have commented on this in the text pointing out to readers that although “Corticosteroid use in TBM is commonplace, dexamethasone is commonly used as it is affordable and widely available although the optimal corticosteroid preparation, dose, and route of administration are unknown.” We have added text to further clarify this point in a sentence following this.

**Chapter Aspirin:**

 The chapter is well written. In addition to the comprehensive antiagregant effect of aspirin, can the author explain how they expect an additional anti-inflammatory effect to high dose dexamethasone?  The duration of the treatment should be discussed.

 Many thanks. These suggestions have been added to this section.

**Chapter Thalidomide:**

 Data on thalidomide are very few and mainly observed in children.

 The authors should be cautious regarding their experience with adjunctive thalidomide in TBM regarding the lack of date. Thalidomide is an old anti-inflammatory drug used in inflammatory diseases and progressively removed from guidelines because of its toxicity. Again, it seems difficult to consider thalidomide as a “promising” drug for TBM since new biological agents are far more efficient on proinflammatory cytokines.

 The authors should not suggest using thalidomide at a large scale in TBM/TB brain abscess.

 Many thanks for your comments on this. We feel differently that the use of thalidomide is supported by now numerous published studies, the most recent being the largest cohort of adult or pediatric patients treated with adjunctive thalidomide for CNS TB–related complications. In this study (reference below), published this year in Clinical Infectious Diseases, thalidomide appeared to be safe, well tolerated and clinically efficacious. Although we appreciate that further RCT generated evidence is required to warrant its use widescale, it is the opinion of the authors on the manuscript that this is indeed a ‘promising’ HDT that warrants discussion and further explanation.

 Ronald van Toorn, Regan S Solomons, James A Seddon, Johan F Schoeman. Thalidomide Use for Complicated Central Nervous System Tuberculosis in Children: Insights From an Observational Cohort. *Clinical Infectious Diseases*, Volume 72, Issue 5, 1 March 2021, Pages e136–e145

**Chapter Immunomodulatory HDT:**

 This chapter is quite short regarding the perspective given by anti-TNF in a severe form of TBM/CNS TB. The choice of the type of anti-TNF, as well as the duration of treatment and the risk associated to this medication, could be discussed. To my mind, anti-TNF therapies may represent one of the best approaches to reduce mortality and morbidity of TBM/TB Abssess in HIV and non HIV people and in children. The main issues are the toxicity of these drugs in the environment where TBM occurs and the availability of these drugs in poor income countries. These points have to be discussed in this review.

 In table 2, the authors should add the following references about adalimumab:

 Adalimumab treatment may replace or enhance the activity of steroids in steroid-refractory tuberculous meningitis.  Lee HS, Lee Y, Lee SO, Choi SH, Kim YS, Woo JH, Kim SH. J Infect Chemother. 2012 Aug;18(4):555-7

 Adalimumab for Corticosteroid and Infliximab-Resistant Immune Reconstitution Inflammatory Syndrome in the Setting of TB/HIV Coinfection.  Lwin N, Boyle M, Davis JS. Open Forum Infect Dis. 2018 Jan 30;5(2):ofy027

 Many thanks for these comments. These references have been added as suggested.

**ART:**

 It is not really fair to consider ART as HDT… even if it is obvious that ART has a significant impact on immunological status. HIV coinfection raises other issues in the management of TBM and I would consider separating this chapter from HDT.

 Many thanks for these comments. As per your suggestion we have separated this section as a distinct sub-chapter within the manuscript.

**Chapter Future Pathways:**

 Chapter Statin therapy - It is not well balanced to have a chapter statin with no data on TBM longer than the chapter on biological agents, even if the background is interesting. I would summarize the impact of statin on multiple sclerosis,  brain injury, etc since it is quite far from TBM to focus on the potential interest of statins on TB and immune response.

 Thank you for these comments, we have shortened this paragraph as suggested.

 FIGURE 2: Not really helpful in the present form.

 I would perform a central plot with increased neuronal survival, immune modulation, axon plasticity myelination that are the goal of treatment; and to describe around the central plot the different pathways to reach this target. 

 Many thanks for your thoughts on this. We agreed with these and attempted to modify the figure as you suggested. However, on review, given that the effect of manipulation of these pathways has not yet been explored in this context we did not want to develop a figure which may be misleading given the early stages of research in this area. We have rather simplified the diagram as a reference figure for readers as they read this section, allowing them to visualise the pathways being discussed.  

**Chapter Host response:**

 The discussion regarding the LTA4H gene and the response to dexamethasone is an important point of pharmacogenetic. The authors should discuss if this strategy is affordable in every setting.

 Many thanks, we agree this is an important issue; we have added a comment on this to the section.
